# Utilization of aqueous distillate and solid residue fractions derived from zero-waste Hydrodistillation of *Zanthoxylum myriacanthum* wall. ex Hook. f. for improving broiler immunity and intestinal functions

**DOI:** 10.1016/j.psj.2026.106952

**Published:** 2026-04-16

**Authors:** Yu-Lei Wang, Chompunut Lumsangkul, Sarana Rose Sommano, Tossapol Moonmanee, Sureerat Numee, Chiao-Hsu Ke, Patipan Hnokaew, Kiattisak Huanhong, Raktham Mektrirat

**Affiliations:** a Veterinary Academic Office, Faculty of Veterinary Medicine, Chiang Mai University, Muang, Chiang Mai, 50100, Thailand; b Department of Animal and Aquatic Sciences, Faculty of Agriculture, Chiang Mai University, Chiang Mai, 50200, Thailand; c Department of Animal Science, National Chung Hsing University, Taichung 40227, Taiwan; d The iEGG and Animal Biotechnology Research Center, National Chung Hsing University, Taichung, 40227, Taiwan; e Department of Plant and Soil Science, Faculty of Agriculture, Chiang Mai University, Chiang Mai, 50200, Thailand; f Department of Veterinary Medicine, School of Veterinary Medicine, National Taiwan University, Taipei City 10617, Taiwan; g Chinese-Thai Cooperation Laboratory of Traditional Chinese Veterinary Medicine and Techniques, Faculty of Veterinary Medicine, Chiang Mai University, Chiang Mai, 50100, Thailand; h Center of Excellence in Pharmaceutical Nanotechnology, Faculty of Pharmacy, Chiang Mai University, Chiang Mai, 50200, Thailand

**Keywords:** Broiler, Feed additive, Gut health, Immunostimulant, Phytobiotic

## Abstract

Phytogenic feed additives derived from plant processing by-products are increasingly explored as sustainable approaches to enhance poultry health, food quality, and production efficiency, supporting food security and sustainable agriculture. This study was to evaluate the effects of dietary supplementation with aqueous distillate (AD) and solid residue (SR) derived from the hydrodistillation of *Zanthoxylum myriacanthum* Wall. ex Hook. f. on safety, meat quality, intestinal morphology, immune responses, and gut microbiota in broiler chickens. A total of 150 one-day-old Ross 308 broilers were randomly assigned to three dietary treatments for 35 days: a basal diet (control), a basal diet supplemented with 1,000 mg/kg AD, or a basal diet supplemented with 1,000 mg/kg SR. For supporting animal health and well-being, neither fraction induced adverse effects on serum biochemical parameters or mortality. No significant differences were observed over the entire experimental period. Both AD and SR significantly improved breast meat redness and affected thawing loss in thigh meat (*p* < 0.05). SR supplementation also significantly increased villus height, and upregulated *GLUT2* expression (*p* < 0.05). Additionally, the expression of *GLUT2* exhibited a significant positive correlation with villus height (*r* = 0.998; *p* = 0.035) and a strong positive association with meat redness (*r* = 0.913; *p* = 0.267). Moreover, the expression of *IL-1β* and *TNF-α* was significantly suppressed by supplementation with both fractions (*p* < 0.05), whereas *IL-10* expression was significantly enhanced by AD supplementation (*p* < 0.05). For gut microbiota analysis, overall microbial diversity was not significantly altered by the supplementations, although the genera *Bacteroides* were significantly altered in both AD and SR groups compared with the control group (*p* < 0.05). These findings support the practical use of hydrodistillation by-products of *Z. myriacanthum* as resource-efficient and environmentally responsible feed additives that promote sustainable production systems, and further dose–response evaluation is recommended to optimize their application in broiler production.

## Introduction

The demand for affordable, high-quality poultry meat is increasing, particularly in nations undergoing development. As a result, per-capita poultry meat consumption is expected to rise substantially in the near future (Kleyn et al., 2021). To optimize poultry farming practices and assure food security, the biological well-being of broiler flocks needs to be maintained within appropriate ranges. Furthermore, the poultry industry has taken up mainly intensive farming practices which require broilers growing efficiently. This is frequently rendered available by in-feed antimicrobial growth promoters, that can lead to drug residues in chicken meat and antimicrobial resistance (Hedman et al., 2020; Muaz et al., 2018). In recent years, due to the widespread occurrence of antimicrobial-resistant bacteria in food-producing animals, the misuse and excessive use of many antibiotics have been limited (Caneschi et al., 2023; Salam et al., 2023). Additionally, the misuse and excessive use of many antibiotics have been restricted due to the increasing prevalence of antimicrobial-resistant bacteria in food-producing animals in recent decades. Consequently, antibiotic-free alternative strategies to enhance flock health and improve growth performance are of considerable interest to the poultry industry.

Available evidence indicates that medicinal plants and natural products exhibit a favorable safety profile and promising potential for improving poultry health and productivity (Dardouri et al., 2025; Rafeeq et al., 2023). Recent investigations have further highlighted the efficacy of various botanical powders, such as garlic, ginger, moringa, and licorice, in modulating growth performance, hematological parameters, and serum lipid profiles in broiler chickens(Kairalla et al., 2022; Kairalla and Alshelmani, 2025, 2022; Kairalla et al., 2023). This underlines the possible benefits of phytogenic dietary supplementation as an appropriate alternative to conventional antibiotics in the feeding of poultry (Abd El-Hack et al., 2022; Frahtia and Derouiche, 2024). Interestingly, previous studies on *Zanthoxylum* species used in poultry have demonstrated multiple functional effects across different applications, including supplementation with *Z. schinifolium* leaf in Sanhuang chicken (Zhang et al., 2025), *Z. bungeanum* leaf in Lohmann Pink laying hens (Cao et al., 2025) and broilers(Chen et al., 2024). In addition, hydrodistillation has been conventionally employed to isolate essential oils from aromatic plants, including *Zanthoxylum* species. Unfortunately, large-scale essential oil production generates substantial amounts of residual water and solid plant residues following hydrodistillation (Rafya et al., 2024). These byproducts frequently retain non-volatile bioactive compounds and other residual constituents, and their improper disposal can result in environmental concerns (Hassouni et al., 2019; Rodrigues et al., 2022). Furthermore, the resulting residues may serve as sources of bioactive substances for diverse applications, including biopesticides, food additives, pharmaceuticals, and cosmetics (Almeida et al., 2024).

To reduce environmental impact, hydrodistillation byproducts are increasingly recognized as valuable sources of biologically active ingredients for applications in the poultry feed supplement industry. Accordingly, the aqueous distillate and solid residues produced during hydrodistillation of *Z. myriacanthum* represent functional byproducts with potential biological activity in feed formulations. Therefore, this study systematically investigated the effects of *Z. myriacanthum* hydrosol and residue on broiler growth performance, meat quality, the expression of immune- and intestinal barrier-related genes, and gut microbiota, providing insights into the potential applications of these byproducts in sustainable poultry farming.

## Materials and methods

### Plant material and extraction

Fruit specimens of *Z. myriacanthum* Wall. ex Hook. f. was harvested from a local farm in Chiang Mai, Thailand (18°47′46.1148″ N, 98°58 45.3468″ E). The specimens were confirmed by a plant taxonomist, and a voucher specimen was deposited in the Bioactive Compound Laboratory Herbarium, Faculty of Agriculture, Chiang Mai University. According to the IUCN Red List of Threatened Species, *Z. myriacanthum* is classified as Least Concern; thus, plant collection complied with IUCN policies and the Convention on International Trade in Endangered Species of Wild Fauna and Flora (CITES).

### Preparation of aqueous distillate and solid residue fractions

The plant material was air-dried and stored at ambient temperature until use. A 250-gram portion of the dried fruits was manually crushed and immersed in 1,500 mL of distilled water prior to hydrodistillation. The extraction was conducted using a heating mantle (MS-E106, Mtops, Kyunggi-do, Korea). The mixture was gradually heated to 100°C and maintained at this temperature for 1 hour. The hydrosol was collected and concentrated using a rotary evaporator, followed by freeze-drying to obtain the aqueous distillate (AD). The solid residue (SR) was collected and air-dried at room temperature.

### Animal husbandry and feeding

All experimental procedures were approved by the Animal Ethics Committee of the Faculty of Agriculture, Chiang Mai University (RAGIACUC013/2567), and all efforts were made to minimize bird discomfort. The study was conducted at the Agriculture Innovation Research, Integration, Demonstration and Training Center, Chiang Mai University, using a completely randomized block design. A total of 150 one-day-old Ross 308 broiler chicks were randomly allocated to 15 floor pens bedded with rice husks, with 10 birds per pen, providing five replicates per treatment. The experimental diets consisted of a basal diet supplemented with 0 mg/kg (control), 1,000 mg/kg aqueous distillate (AD), or 1,000 mg/kg solid residue (SR). The nutrient composition of the basal diets for the starter (1–21 d) and finisher (22–35 d) phases is presented in Table 1. Feed and water were provided ad libitum throughout the 35-d experimental period. Birds were reared at a stocking density of 7 birds/m² in a conventional open-sided house equipped with side curtains and ventilation fans. Environmental conditions were maintained at 20 ± 3°C, relative humidity of 65–75%, with a 12-h photoperiod.

**Table 1:** Chemical composition of the commercial broiler diet^1^.

Ingredients (%)	Basal diet	
	Starter diets (1-21 days)	Finisher diet (22-35 days)
Corn	55.50	57.50
Soybean meal	35.00	33.50
Fish meal	3.00	2.00
Soybean oil	2.00	3.00
Salt	0.35	0.35
Dicalcium phosphate	1.20	1.10
Limestone	1.20	1.00
L-lysine.HC1	0.20	0.15
DL-methionine	0.28	0.28
Vitamin premix	0.50	0.50
Mineral premix	0.50	0.50
Proximate composition (Calculated)		
Dry matter (% of feed)	90.18	90.33
Crude protein (% of DM)	21.92	20.83
Ether extract (% of DM)	4.87	5.85
Crude fiber (% of DM)	2.45	2.44
ME (kcal/kg)	3020	3110

1 Premix: The supplied vitamin-–mineral premix contains the following per kilogram of diet: 15,000 IU vitamin A, 3000 IU vitamin D3, 25 IU vitamin E, 5 mg vitamin K3, 2 mg vitamin B1, 7 mg vitamin B2, 4 mg vitamin B6, 25 mg vitamin B12, 11.4 mg pantothenic acid, 35 mg nicotinic acid, 1 mg folic acid, 15 μg biotin, 250 mg choline chloride, 1.6 mg Cu, 60 mg Mn, 45 mg Zn, 80 mg Fe, 0.4 mg I, and 0.15 mg Se.

### Performance measurements

Body weight (BW) of broilers was recorded on days 1, 7, 14, 21, 28, and 35 of the experimental periods. Daily feed intake was continuously monitored on a pen basis, and these data were used to calculate average daily gain (ADG), average daily feed intake (ADFI), and feed conversion ratio (FCR) according to standard poultry production formulas.

### Sample collection

On day 35, after a 12-h fasting period during which water was provided ad libitum, birds were processed for blood sample collection. Brachial venipuncture was performed on 15 randomly selected birds per group. Chickens were euthanized by gradual-fill CO₂ inhalation at 30% chamber volume per minute, followed by cervical disarticulation. Death was confirmed by the absence of a heartbeat and pupillary light reflex prior to tissue collection. Three chickens with body weights close to the group mean were selected from each treatment for cecal content sampling, and gut microbiota were analyzed by 16S rRNA gene sequencing. Additionally, jejunal samples were collected from six chickens per treatment to assess immune- and intestinal barrier–related gene expression. Samples of the duodenum, jejunum, and ileum were collected for intestinal morphology analysis.

### Serum biochemistry

Serum from blood was separated by centrifugation at 2200 × *g* for 15 min following stabilization for 30 min at room temperature, and then samples were immediately stored at −80°C for serum biochemical analysis. The levels of alanine aminotransferase (ALT), alkaline phosphatase (ALP), aspartate aminotransferase (AST), total bilirubin (TBIL), direct bilirubin (DBIL), indirect bilirubin (IBIL), total protein (TP), albumin (ALB), and globulin (GLB) in serum samples were analyzed using a Sysmex BX-3010 automated analyzer (Kobe, Japan).

### Carcass and organ measurements

From each broiler, the heart, liver, gizzard, spleen, giblets, and abdominal fat samples were weighed separately and expressed as a percentage of body weight. Then, the carcass yield percentage was estimated based on the eviscerated weight relative to pre-slaughter live weight and yields from each carcass part were calculated as a proportion of the eviscerated weight.

### Meat physicochemical analysis

The breast and thigh muscles were collected, and their pH, color, quality, and nutritional composition were assessed, each measured in triplicate. pH values at 45 min and 24 h postmortem was measured using a pH meter (HI98163, Hanna Instruments, RI). Color values (L*, a*, and b*) were measured using a colorimeter (CR-400, Konica Minolta, Japan). Six replicates were analyzed for each treatment. The moisture, protein, and fat contents were analyzed following AOAC methods (Thiex, 2009). Following carcass evaluation, meat samples were refrigerated at 4°C for 24 h prior to assessment of water-holding capacity, including drip loss and thawing loss, as will as shear force, using standard procedures (Honikel, 1998). To determine drip loss (%), meat samples were sealed in plastic bags and stored at 4°C for 24 h; weight loss was calculated as the percentage difference between initial and final weights. To determine thawing loss (%), the frozen meat samples were thawed at 4°C for 24 h; after being blotted dry with absorbent paper, the samples were reweighed, and the weight loss was calculated as a percentage of the initial frozen weight. The meat samples were allowed to cool to room temperature. Rectangular strips (1 × 1 × 2 cm) were excised parallel to the longitudinal orientation of the muscle fibers. Shear force was measured perpendicular to the fiber axis using a TA-XT2 texture analyzer (Stable Micro Systems, Godalming, UK) equipped with a Warner-Bratzler blade (HDP/BS).

### Intestinal histomorphometry

Intestinal samples from the duodenum, jejunum, and ileum collected on day 35 were fixed in 4% paraformaldehyde and dehydrated through a graded alcohol series. The tissues were then embedded in paraffin, sectioned, and stained with hematoxylin and eosin. Histological images were captured using a Nikon microscope at 10 × 20 magnification. Villus height (VH) and crypt depth (CD) were measured using Image-Pro Plus 6.0 software, and the villus height–to–crypt depth (VH:CD) ratio was calculated.

### Assessments of immune- and intestinal function–related gene expression

Total RNA was extracted from jejunal tissues using the PureLink® RNA Mini Kit (Invitrogen, Thermo Fisher Scientific), and RNA concentration and purity were determined by spectrophotometry using a NanoDrop 1000 (Thermo Fisher Scientific). Gene-specific primers were designed to target immune-related genes, host defense–related peptides, tight junction and intestinal barrier function–related genes, and nutrient-sensing genes, as listed in Table 2. β-Actin was used as the housekeeping gene (Li et al., 2014). Quantitative real-time PCR was performed using the qPCRBIO SyGreen® 1-Step Go Lo-ROX kit (PCR Biosystems, London, UK) on a StepOnePlus™ Real-Time PCR System (Applied Biosystems). Each reaction was conducted in a final volume of 20 μL, consisting of 10 μL of 2 × qPCRBIO SyGreen 1-Step Mix, 0.8 μL each of forward and reverse primers (10 μM), 1 μL of 20 × RTase Go, 10 ng of total RNA, and nuclease-free water. The thermal cycling conditions were as follows: initial hold at 42°C for 2 min, reverse transcription at 45°C for 10 min, polymerase activation at 95°C for 2 min, followed by 40 cycles of denaturation at 95°C for 5 s and annealing/extension at 65°C for 30 s. Melt curve analysis was performed at 95°C for 15 s, 60°C for 1 min, 95°C for 15 s, and 60°C for 15 s. Relative gene expression levels were calculated using the 2⁻ΔΔCt method with β-actin as the internal control, and amplification efficiency was verified using a standard curve. (Livak and Schmittgen, 2001).

**Table 2:** Primer sequences used for quantitative real-time PCR analysis of housekeeping, gut function–related and immune-related genes in broiler jejunum.

Target gene	Primer Sequences	Accession No.
Housekeeping gene		
β-actin	F: CTGGCACCTAGCACAATGAA R: ACATCTGCTGGAAGGTGGAC	X00182.1
Gut function–related genes		
OCLDN	F: ACGGCAGCACCTACCTCAA R: GGCGAAGAAGCAGATGAG	NM_205128.1
CLDN1	F: TGGAGGATGACCAGGTGAAGA R: CGAGCCACTCTGTTGCCATA	NM_001013611.2
TJAP1	F: AGGAAGCGATGAATCCCTGTT R: TCACTCAGATGCCAGATCCAA	421455
MUC2	F: ATGCGATGTTAACACAGGACTC R: GTGGAGCACAGCAGACTTTG	JX284122.1
GLUT2	F: AGATGACAGCTCGCCTGATG R: GTCTTCAATCACCTTCTGCGG	396130
Immune-related genes		
IL-1β	F: GTGAGGCTCAACATTGCGCTGTA R: TGTCCAGGCGGTAGAAGATGAAG	Y15006
TNF-α	F: TGCTGTTCTATGACCGCC R: CTTTCAGAGCATCAACGCA	NM204267
IL-12	F: CTGAAGGTTGCAGAAGCAGAG R: CCAGCTCTGCCTTGTAGGTT	NM213588
IL-10	F: AGCAGATCAAGGAGACGTTC R: ATCAGCAGGGTACTCCTCGAT	NM_001004414.4
AvBD1	F: AAACCATTGTCAGCCCTGTG R: TTCCTAGAGCCTGGGAGGAT	395841
CATHL2	F: AGGAGAATGGGGTCATCAGG R: GGATCTTTCTCAGGAAGCGG	420407

**Footnotes:** F, forward primer; R, reverse primer. Housekeeping gene: β-actin (ACTB). Gut function–related genes: OCLN, occludin; CLDN1, claudin-1; TJAP1, tight junction–associated protein 1; MUC2, mucin-2; GLUT2, glucose transporter 2. Immune-related genes: IL-1β, interleukin-1 beta; IL-10, interleukin-10; IL-12, interleukin-12; TNF-α, tumor necrosis factor alpha; AvBD1, avian beta-defensin 1; CATHL2, cathelicidin 2. Accession numbers correspond to sequences obtained from GenBank.

### Sequencing of 16S rRNA and bioinformatics

RNA was extracted from cecal contents using the ZymoBIOMICS®-96 MagBead RNA Kit (Zymo Research, Irvine, CA) on an automated platform. The concentration and purity of RNA were assessed via spectrophotometry (Nanodrop ND1000; Thermo Scientific). Bacterial 16S ribosomal RNA genes were sequenced with the Quick-16S™ NGS Library Prep Kit (Zymo Research, Irvine, CA). The V3-V4 regions of the 16S rRNA gene were amplified through polymerase chain reaction (PCR) using primers 338F (5′-ACTCCTACGGGAGGCAGCAG-3′) and 806R (5′-GGACTACHVGGGTWTCTAAT-3′) on an ABI GeneAmp 9700 PCR thermocycler (Thermo Fisher Scientific). Real-time PCR was performed during the library preparation. The final PCR products were quantified using quantitative PCR (Qpcr) and pooled based on their molar concentrations. The library was cleaned using the Select-a-Size DNA Clean & Concentrator™ (Zymo Research, Irvine, CA), and concentrations of PCR products were measured using TapeStation® (Agilent Technologies) and Qubit® (Thermo Fisher Scientific). Unique amplicon sequence variants from raw reads were identified using the DADA2 pipeline (Callahan et al., 2016) after filtering for sequencing errors and chimeras. Sequences were taxonomically classified using Uclust in Qiime v.1.9.1 with the Zymo Research 16S database as the reference. The composition, alpha-diversity, and beta-diversity were analyzed in QIIME v.1.9.1(Caporaso et al., 2010).

### Statistical analysis

Replicate served as the experimental unit, and data were analyzed using descriptive statistics. Normality was assessed using the Shapiro–Wilk test and Q–Q plots. Differences among treatments were analyzed by one-way ANOVA using the GLM procedure, followed by Tukey’s multiple comparison test. Statistical analyses were performed using SAS software (SAS Institute Inc., Cary, NC). Statistical significance was declared at *p* < 0.05. Spearman’s rank correlation analysis was used to assess relationships among variables. Figures were generated using GraphPad Prism 10 (GraphPad Software, San Diego, CA, USA).

## Results

### Growth performance

The effects of dietary supplementation with *Z. myriacanthum* fractions on the growth performance of broiler chickens are presented in Table 3. During the early growth phase (0-21 days), significant differences were observed in body weight (BW), ADG, and FCR. Chickens in the control group had the highest mean BW (*p* < 0.05) and ADG (*p* < 0.05), with these values progressively decreasing in the AD and SR groups. Although ADFI was similar across all groups *(p* = 0.57), FCR differed significantly. In addition, the control group exhibited the lowest FCR (1.59), whereas the AD group showed the highest FCR (1.68) (*p* = 0.0018). However, no significant differences were observed among the experimental groups for any parameter during the second growth phase (21–35 days) or across the entire experimental period (0–35 days) (*p* > 0.05).

**Table 3:** Growth performance of broiler chickens fed a control diet or diets supplemented with *Zanthoxylum myriacanthum* fractions during different growth phases.

Items	Experimental groups			SEM	*p*-value
	Control	AD	SR		
Starter phase (0–21 d)					
BWG, g	785.98^a^	750.28^b^	731.55^b^	7.269	0.0007
ADG, g/d	37.42^a^	35.94^b^	34.84^c^	0.329	0.0005
ADFI, g/d	59.48	59.13	58.73	0.388	0.4530
FCR, g/g	1.59^b^	1.63^a^	1.68^a^	0.014	0.0018
Finisher phase (22–35 d)					
BWG, g	1239.92	1266.41	1283.03	38.423	0.7490
ADG, g/d	88.56	90.46	91.65	2.745	0.7486
ADFI, g/d	164.60	161.99	160.71	1.501	0.5159
FCR, g/g	1.88	1.79	1.78	0.063	0.5478
Whole phase (0–35 d)					
BWG, g	2025.89	2025.90	2035.13	44.371	0.9877
ADG, g/d	57.88	57.88	57.54	1.230	0.9779
ADFI, g/d	101.38	100.27	101.04	0.604	0.4749
FCR, g/g	1.76	1.73	1.76	0.038	0.8935

Abbreviations: AD, basal diet supplemented with 1,000 mg/kg aqueous distillate; SR, basal diet supplemented with 1,000 mg/kg solid residue; BWG, body weight gain; ADG, average daily gain; ADFI, average daily feed intake; FCR, feed conversion ratio; and SEM, standard error of the mean. Values are presented as means. Different superscript letters (a, b) within the same row indicate significant differences (*p* < 0.05).

### Serum biochemical profiles

For safety evaluation of dietary supplementation with *Z. myriacanthum* fractions, no fatalities occurred in any animal over the 35-day period. No discernible changes in general appearance or behavior were observed throughout the research period. For serum biochemical parameters of broiler chickens is summarized in Table 4. Significant differences were observed between the basal diet group and the SR-supplemented groups in total protein, albumin, and globulin levels. However, supplementation with *Z. myriacanthum* fractions did not affect lipid parameters or serum enzyme activities, including aspartate transaminase, alanine transaminase, and alkaline phosphatase (*p* > 0.05).

**Table 4:** Serum biochemical profiles of broiler chickens fed a control diet or diets supplemented with *Zanthoxylum myriacanthum* fractions at 35 days of age.

Items	Experimental groups				
	Control	AD	SR	SEM	*p-*value
Total cholesterol (mg/dL)	98.66	125.30	103.70	7.386	0.0828
Triglyceride (mg/dL)	56.50	50.67	48.00	7.290	0.7703
Aspartate transaminase(U/L)	375.50	338.00	358.70	44.978	0.8694
Alanine transaminase (U/L)	1.50	0.99	1.33	0.281	0.5304
Alkaline phosphatase (U/L)	1602.50	1448.00	1810.00	117.232	0.1867
Total protein (g/dL)	3.50^a^	3.23^ab^	2.80^b^	0.112	0.0248
Albumin (g/dL)	1.25^a^	1.30^a^	1.03^b^	0.046	0.0209
Globulin (g/dL)	2.25^a^	1.93^ab^	1.77^b^	0.081	0.0341
Total bilirubin (g/dL)	0.04	0.09	0.07	0.031	0.6149
Direct bilirubin (g/dL)	0.02	0.04	0.02	0.005	0.1317
Indirect bilirubin (g/dL)	0.02	0.05	0.04	0.032	0.8135

Abbreviations: AD, basal diet supplemented with 1,000 mg/kg aqueous distillate; SR, basal diet supplemented with 1,000 mg/kg solid residue; and SEM, standard error of the mean. Values are presented as means. Different superscript letters (a, b) within the same row indicate significant differences (*p* < 0.05).

### Carcass traits

Carcass characteristics and organ weights of broilers fed a basal diet and diets supplemented with aqueous distillate or solid residues are summarized in Table 5. The live weight and eviscerated weight did not differ significantly among groups, and the carcass yield was not affected by dietary treatment (*p* = 0.38). Regarding organ composition, the AD and SR groups showed no effects on gizzard, heart, or spleen weights. The liver and intestine weight percentages in the control group were higher than those in the AD group (*p* < 0.05). The intestine weight percentage in the control group was also higher than that in the SR group (*p* < 0.05). For the percentage of retail cold carcass weight, most carcass components did not differ significantly among groups. The head weight percentage in the AD group was lower than that in the SR and control groups (*p* < 0.05).

**Table 5:** Carcass yield and characteristics of broiler chickens fed a control diet or diets supplemented with *Zanthoxylum myriacanthum* fractions.

Items	Experimental groups			SEM	*p*-value
	Control	AD	SR		
Live weight (g)	2054.4	2029.5	2032	83.85	0.8800
Eviscerated weight (g)	1747.81	1734.8	1729.59	20.366	0.8327
Carcass yield (%)	84.68	85.47	85.11	0.41	0.3800
Organ of composition (%)					
Liver	2.57^a^	2.30^b^	2.48^ab^	0.067	0.0373
Gizzard	2.74	2.62	2.59	0.122	0.6849
Heart	0.60	0.62	0.63	0.025	0.5947
Spleen	0.14	0.12	0.14	0.018	0.6659
Intestine	4.94^a^	3.83^c^	4.33^b^	0.163	0.0006
Retail cut yields (%)					
Head	3.07^a^	2.62^b^	3.15^a^	0.0998	0.0022
Neck	6.06	5.42	4.95	0.3778	0.1674
Feet	4.35	4.51	4.5	0.1335	0.6826
Breast	28.19	28.83	28.31	0.7130	0.8077
Thighs	12.54	12.50	12.14	0.3697	0.7224
Drumsticks	12.18	12.33	12.74	0.2522	0.3082
Wings	9.20	9.45	9.80	0.3032	0.4115
Skeleton	24.49	24.54	24.76	0.7447	0.9652

Abbreviations: AD, basal diet supplemented with 1,000 mg/kg aqueous distillate; SR, basal diet supplemented with 1,000 mg/kg solid residue; and SEM, standard error of the mean. Values are presented as means. Different superscript letters (a, b) within the same row indicate significant differences (*p* < 0.05).

### Meat quality

The effects of diets supplemented with *Z. myriacanthum* fractions on the physicochemical properties of broiler meat are summarized in Table 6. The results demonstrated that AD and SR supplementation did not affect pH at 45 min postmortem, drip loss, shear force, or moisture content in either breast or thigh meat. For breast meat, muscular redness and fat content in the AD and SR groups were higher than those in the control group (*p* < 0.05), whereas pH at 24 h postmortem in both supplemented groups was lower than that in the control group (*p* < 0.05). Breast protein content in the control and SR groups was significantly higher than that in the AD group (*p* < 0.05). For thigh meat, pH at 24 h postmortem was also significantly affected by supplementation with both fractions (*p* < 0.05), and supplementation with both fractions significantly affected thawing loss compared with the basal diet (*p* < 0.05).

**Table 6:** Physicochemical properties and nutrient composition of breast and thigh meat from broiler chickens fed a control diet or diets supplemented with *Zanthoxylum myriacanthum* fractions.

Items	Experimental groups			SEM	*p-*value
	Control	AD	SR		
Breast					
pH_45_	6.69	6.66	6.79	0.0585	0.2645
pH_24_	6.03^a^	5.86^b^	5.85^b^	0.0294	<.0001
Lightness, L*	56.53	57.56	57.90	0.5602	0.2239
Redness, a*	−0.71^a^	−0.18^b^	0.01^b^	0.1376	0.002
Yellowness, b*	10.58	11.89	12.10	0.4611	0.0577
Drip loss (%)	2.63	2.23	2.30	0.2967	0.6544
Thawing loss (%)	15.24	14.75	16.65	1.6092	0.6961
Shear force (N)	32.40	33.83	34.72	4.3489	0.9306
Moisture (%)	73.94	75.28	75.16	0.6288	0.3738
Protein (%)	24.05^a^	23.40^b^	24.10b^a^	0.1041	0.0299
Fat (%)	4.84^b^	7.71^a^	6.43^a^	0.3045	0.0159
Thigh					
pH_45_	6.76	6.79	6.73	0.0522	0.677
pH_24_	6.68^a^	5.85^c^	6.38^b^	0.0279	<.0001
Lightness, L*	57.50	56.29	56.67	0.8543	0.6166
Redness, a*	0.73	0.84	0.66	0.2073	0.8307
Yellowness, b*	8.63	8.70	8.19	0.5973	0.8108
Drip loss (%)	3.47	2.61	2.99	0.2435	0.0934
Thawing loss (%)	17.44^a^	13.09^b^	12.67^b^	1.2573	0.0488
Shear force (N)	15.71	18.96	17.67	1.3954	0.2697
Moisture (%)	77.70	76.64	77.70	0.3886	0.1926
Protein (%)	22.55	23.40	22.55	0.2483	0.1865
Fat (%)	12.44	10.97	12.44	1.2947	0.7393

Abbreviations: AD, broilers fed a basal diet supplemented with 1,000 mg/kg aqueous distillate; SR, broilers fed a basal diet supplemented with 1,000 mg/kg solid residue; pH45, pH at 45 min postmortem; pH24, pH at 24 h postmortem; and SEM, standard error of the mean. Values are presented as means. Different superscript letters (a, b) within the same row indicate significant differences (*p* < 0.05).

### Intestinal morphostructural characteristics

The effects of dietary supplementation of *Z. myriacanthum* fractions on the intestinal morphology of broiler chickens are presented in Table 7. Villus height in the jejunum and ileum was significantly greater in the SR group than in the AD and control groups (*p* < 0.05), whereas duodenal villus height in the SR group did not differ from that in either group. Regarding crypt depth, both supplemented groups had significantly shallower duodenal crypts than the control group (*p* < 0.05). In contrast, ileal crypt depth in the control group was shorter than that in both supplemented groups (*p* < 0.05). No significant differences in the VH:CD ratio were observed among treatments in the duodenum, jejunum, or ileum.

**Table 7:** Intestinal morphology of broiler chickens fed a control diet or diets supplemented with *Zanthoxylum myriacanthum* fractions at 35 days of age.

Items	Experimental groups			SEM	*p*-value
	Control	AD	SR		
Duodenum					
Villus hight (μm)	1059.39^a^	898.86^b^	982.22^ab^	50.8794	0.0924
Crypt depth (μm)	169.98^a^	135.76^b^	137.53^b^	9.8026	0.0270
VH:CD	6.84	6.78	7.45	0.4879	0.5690
Jejunum					
Villus hight (μm)	964.36^b^	893.43^b^	1036.26^a^	47.649	0.1156
Crypt depth (μm)	96.80^bc^	93.64^c^	132.87^a^	7.7613	0.0010
VH:CD	8.74	9.41	8.68	0.4261	0.3969
Ileum					
Villus hight (μm)	885.18^b^	936.64^b^	1052.91^a^	26.170	0.0001
Crypt depth (μm)	85.35^b^	110.79^a^	110.87^a^	7.1994	0.0206
VH:CD	11.29	9.21	10.57	0.8115	0.1910

Abbreviations: AD, broilers fed a basal diet supplemented with 1,000 mg/kg aqueous distillate; SR, broilers fed a basal diet supplemented with 1,000 mg/kg solid residue; VH, villus height; CD, crypt depth; VH:CD, villus height–to–crypt depth ratio; and SEM, standard error of the mean. Values are presented as means. Different superscript letters (a, b) within the same row indicate significant differences (*p* < 0.05).

### Gut functional gene expression

**Fig. 1:**
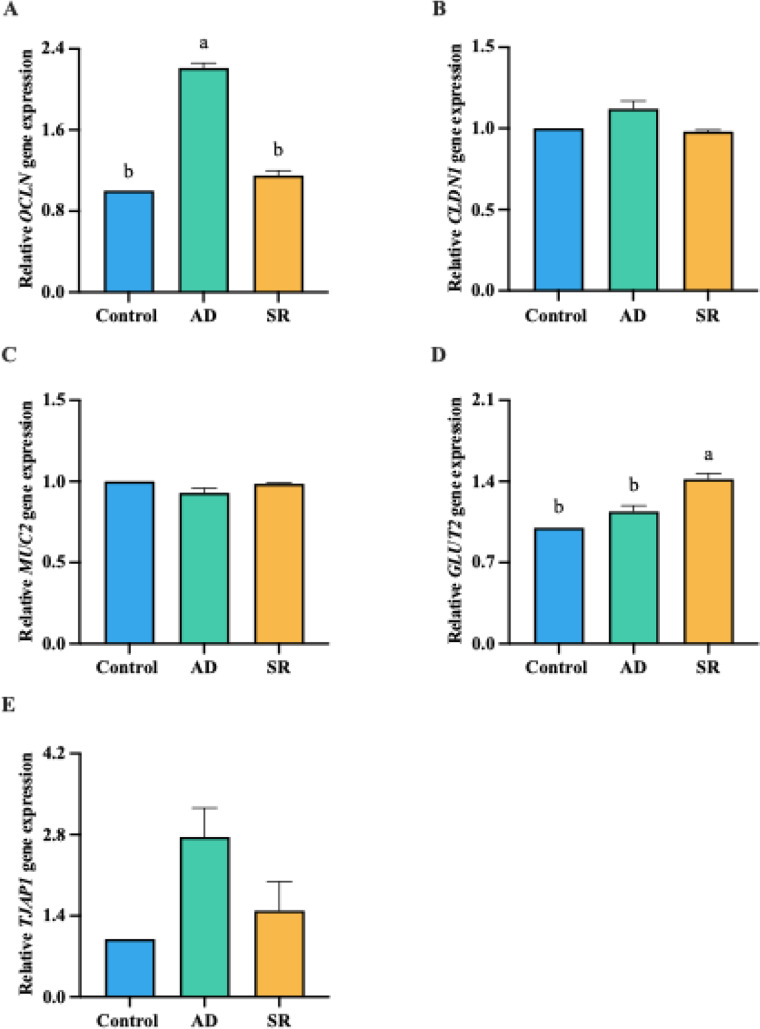
Effects of *Zanthoxylum myriacanthum* fractions on intestinal functional gene expression in the jejunum of broiler chickens fed control, aqueous distillate (AD), or solid residue (SR) diets at 35 days of age. Panels represent (A) OCLN, (B) CLDN1, (C) TJAP1, (D) MUC2, and (E) GLUT2. Data were analyzed using one-way ANOVA followed by Tukey’s multiple comparison test. Different lowercase letters (a, b) indicate significant differences among treatments (*p* < 0.05).

The effects of AD and SR supplementation on broiler intestinal expression of tight junction proteins, nutrient-sensing–related genes, and barrier function–related genes are presented in Fig. 1. The results demonstrated that OCLN expression in the AD group was significantly higher than that in the control and SR groups (*p* < 0.05). TJAP1 showed a similar trend; however, no significant differences were observed among groups. Notably, GLUT2 expression was markedly upregulated in the SR group compared with the control and AD groups (*p* < 0.05). No significant differences were observed in CLDN-1 or MUC2 expression among the experimental groups.

**Fig. 2:**
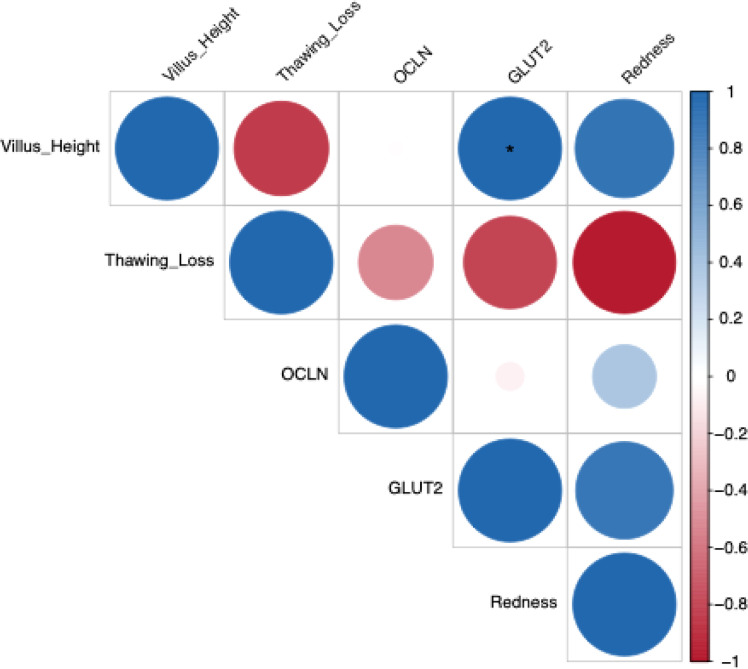
Effects of *Zanthoxylum myriacanthum* fractions on the mechanistic relationships linking intestinal barrier–related gene expression, villus morphostructural indices, and meat quality parameters in broiler chickens. Spearman’s rank correlation correlogram illustrates significant associations among variables, reflecting the potential cascade from gene regulation to intestinal morphology and subsequent meat quality outcomes. Positive correlations are shown in blue and negative correlations in red. Color intensity and circle size indicate the strength of correlation coefficients. Asterisks (*) denote statistically significant correlations (*p* < 0.05).

Spearman’s rank correlation analysis was used to assess relationships among significant variables related to meat quality, intestinal morphostructural indices, and intestinal function, as reflected by the expression of tight junction proteins, nutrient-sensing–related genes, and barrier function–related genes (Fig. 2). The expression of *GLUT2* exhibited a significant positive correlation with VH (*r* = 0.998; *p* = 0.035). In addition, GLUT2 expression showed a strong positive correlation with meat redness (*r* = 0.913; *p* = 0.267). In contrast, *OCLN* expression was negatively correlated with thawing loss (*r* = −0.523; *p* = 0.649). Moreover, thawing loss was negatively correlated with other parameters, including VH (*r* = −0.841; *p* = 0.364), *GLUT2* expression (*r* = −0.810; *p* = 0.399), and meat redness (*r* = −0.979; *p* = 0.123).

### Immune-related gene expression

**Fig. 3:**
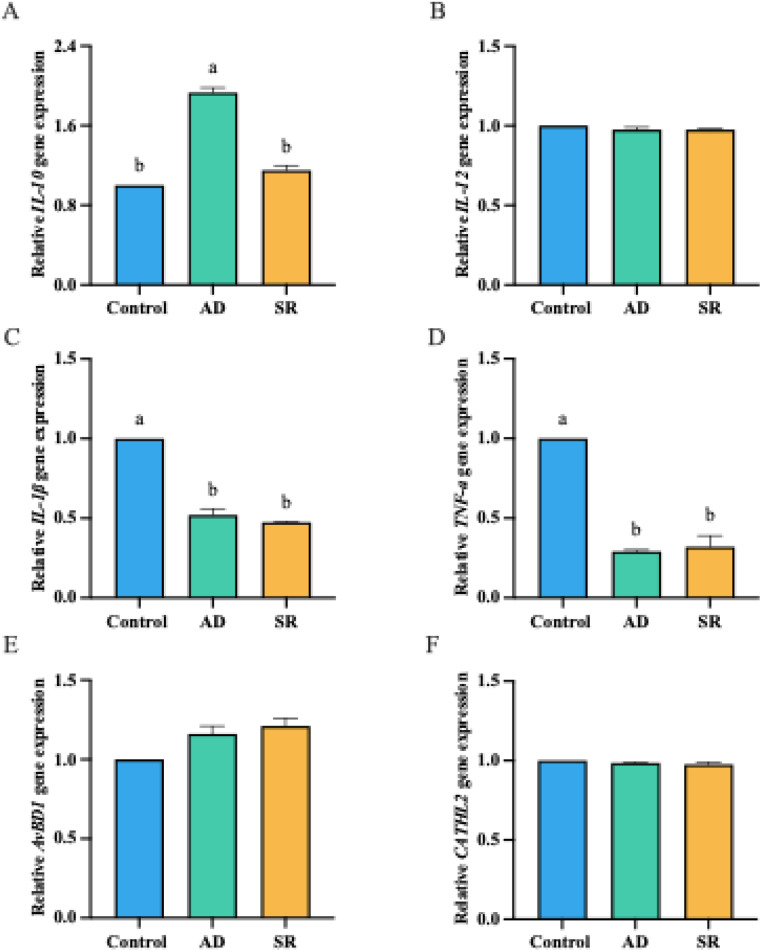
Effects of *Zanthoxylum myriacanthum* fractions on the relative mRNA expression of immune-related genes in the jejunum of broiler chickens fed control, aqueous distillate (AD), or solid residue (SR) diets at 35 days of age. Panels show (A) IL-1β, (B) TNF-α, (C) IL-12, (D) IL-10, (E) AvBD1, and (F) CATHL2. Data were analyzed by one-way ANOVA followed by Tukey’s multiple comparison test. Different lowercase letters (a, b) indicate significant differences among treatments (*p* < 0.05).

Analysis of immune-related gene expression in the jejunum revealed modulatory effects of AD and SR supplementation on pro-inflammatory cytokines, anti-inflammatory cytokines, and host defense peptides (Fig. 3). Among the pro-inflammatory cytokines, *IL-1β* and *TNF-α* expression was significantly reduced in both the AD and SR groups compared with the control group (*p* < 0.05). Moreover, the expression of *IL-10*, an anti-inflammatory cytokine, was significantly upregulated in the AD group compared with the control and SR groups (*p* < 0.05). In contrast, the expression of *IL-12, AvBD1*, and *CATHL2* did not differ significantly among groups.

### Cecal microbial community structure

**Fig. 4:**
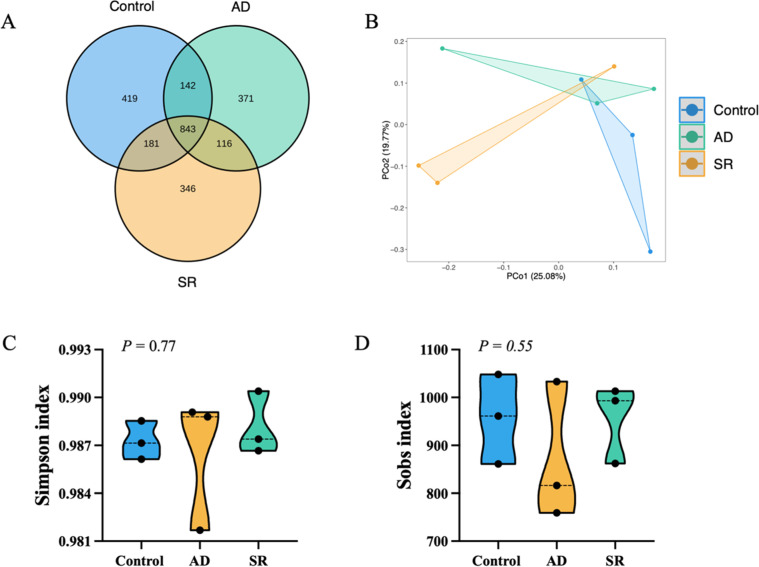
Effects of *Zanthoxylum myriacanthum* fractions on cecal microbiota diversity and community structure in broiler chickens fed control, aqueous distillate (AD), or solid residue (SR) diets. (A) Venn diagram showing shared and unique OTUs among groups. (B) Principal coordinate analysis (PCoA) based on Bray–Curtis dissimilarity. (C) Simpson index and (D) Sobs index for alpha diversity. Statistical differences were evaluated using PERMANOVA for beta diversity and one-way ANOVA for alpha diversity (*p* < 0.05).

Total DNA was extracted from nine cecal samples, comprising three samples from each of the control, AD, and SR-supplemented groups. A total of 843 amplicon operational taxonomic units (OTUs) were shared among the experimental groups. Fig. 4A presents a Venn diagram illustrating the OTUs shared and unique to each group. In addition to the core microbial community, each group possessed a distinct set of unique OTUs: 419 in the control, 371 in the AD, and 346 in the SR group, indicating treatment-specific effects on gut microbial composition***.*** The proportion of core OTUs was higher in the AD (57.26%) and SR (56.73%) groups than in the control (53.19%). Pairwise comparisons revealed partial OTU overlap between groups, indicating functional convergence under similar conditions.

Alpha diversity of the cecal microbiota in broiler chickens was evaluated using the Simpson (Fig. 4C) and Sobs (Fig. 4D) indices. The results indicated that no statistically significant differences in microbial evenness or richness were detected among the experimental groups. The dissimilarity in bacterial community structure was examined through PCoA based on Bray-Curtis dissimilarity (Fig. 4B), and the difference among the three treatments was determined by PERMANOVA. Differences in bacterial community structure among groups were evidenced by the clear separation of communities along the PCoA1 and PCoA2 axes, which together explained 44.85% of the total variation (PCo1: 25.08%; PCo2: 19.77%).

### Discriminatory cecal microbial taxa

**Fig. 5:**
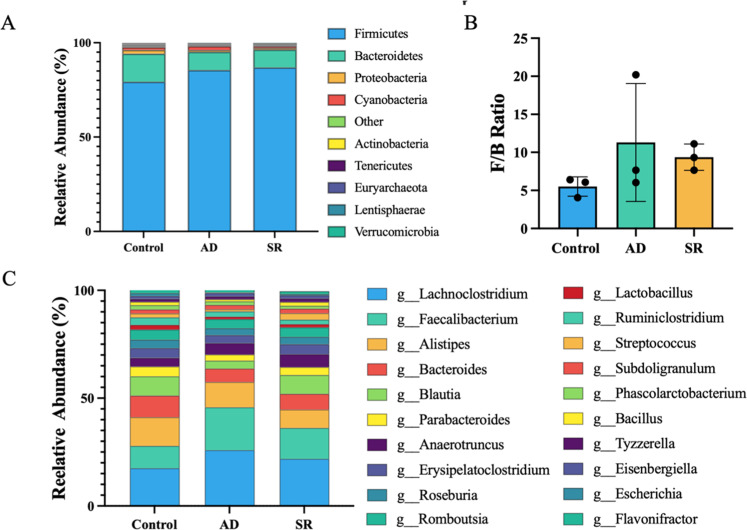
Effects of *Zanthoxylum myriacanthum* fractions on the relative abundance of cecal microbiota in broiler chickens fed control, aqueous distillate (AD), or solid residue (SR) diets. (A) Top 10 phylum-level composition. (B) Firmicutes-to-Bacteroidetes (F/B) ratio. (C) Top 20 genus-level composition of predominant taxa. Data were analyzed using one-way ANOVA (*p* < 0.05).

The relative abundance of the predominant bacterial top 10 phyla and top 20 genera in the ceca is shown in Fig. 5. At the phylum level, Bacteroidetes and Firmicutes were the dominant phyla across all groups, accounting for over 80% of the identified sequences (Fig. 5A). The Firmicutes-to-Bacteroidetes (F/B) ratio in the AD and SR groups showed an increasing trend but did not differ significantly from that of the control group (Fig. 5B). The predominant genera across all treatments were *Lachnoclostridium, Faecalibacterium, Alistipes*, and *Bacteroides*, with no significant differences observed among groups (Fig. 5C).

**Fig. 6:**
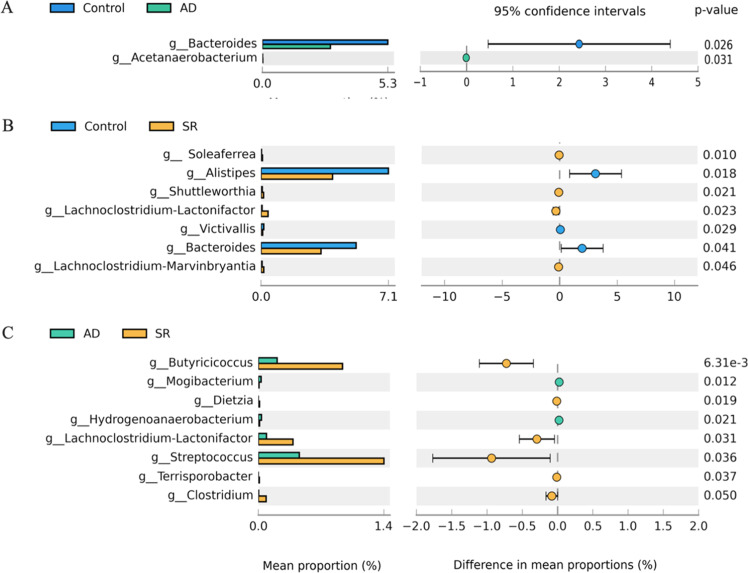
Effects of *Zanthoxylum myriacanthum* fractions on differentially abundant cecal bacterial genera in broiler chickens fed control, aqueous distillate (AD), or solid residue (SR) diets. Genera with significant differences were identified using STAMP based on pairwise comparisons: (A) control and AD groups, (B) control and SR groups, and (C) AD and SR groups. Statistical significance was determined using Welch’s t-test (*p* < 0.05).

Linear Discriminant Analysis Effect Size (LEfSe) was performed to identify microorganisms that significantly discriminated among the three experimental groups. Taxa distinguished in the control group included the class *Betaproteobacteria*, the order *Burkholderiales*, and the families *Alcaligenaceae* and *Bacteroidaceae*. Pairwise comparisons using STAMP demonstrated that the genera *Bacteroides* was significantly altered in both the AD and SR groups compared with the control group (*p* < 0.05). In addition, other bacterial genera significantly affected by AD supplementation are shown in Fig. 6A, whereas those significantly influenced by SR supplementation are presented in Fig. 6B. Furthermore, significant differences in the relative abundance of *Butyricicoccus, Mogibacterium, Diezia, Hydrogenoanaerobacterium, Lachoclostridium-lactonifactor, Streptococcus, Terrisporobacter* and *Clostridium* were observed between the AD and SR groups (Fig. 6C).

## Discussion

The use of plant-derived feed additives in livestock farming is being increasingly recognized as an appropriate and effective alternative to traditional antibiotics. For instance, bioactive compounds in plants such as terpenes, flavonoids, and phenolics have shown antibacterial, antioxidant, and gut health-promoting effects (Luo et al., 2023; Radha et al., 2024). Hydrodistillation has long been used to extract essential oils from medicinal and aromatic plants (Katekar et al., 2023). Previous studies on *Z. myriacanthum* have demonstrated that DL-limonene and sabinene are the dominant constituents, with the hexane extract containing 29.75% DL-limonene and 9.76% sabinene, while the methylene chloride extract was characterized by higher proportions of limonene (40.70%) and sabinene (16.60%) (Kruewong and Auamcharoen, 2023). A hydrolate, or hydrosol, is the aqueous by-product of essential oil distillation containing dispersed oil droplets and water-soluble oxygenated compounds. In. addition, Aqueous distillate composition has predominantly been examined based on its volatile constituents. (Tavares et al., 2022). Despite the removal of volatile oils by hydrodistillation, the solid residue retains non-volatile and semi-volatile phytochemicals, such as phenolic compounds (Skendi et al., 2022). The presence of non-volatile phenanthridine alkaloids in *Z. myriacanthum* has been documented (Sukari et al., 1999). Consequently, the aqueous distillate and solid residues produced during hydrodistillation should not be considered waste, but rather valuable sources of residual bioactive compounds that may contribute to biological activity when incorporated into feed formulations. However, phytochemical profiling of the fractions was not performed in this study; therefore, the proposed mechanisms should be interpreted cautiously and are based on previously reported phytochemical constituents. Further studies are needed to confirm these associations.

Supplementation with AD and SR fractions affected growth performance during the early phase (0–21 days). However, no differences were observed during the later phase or across the overall rearing period, suggesting a transient effect, possibly related to physiological adaptation during early growth. This observation aligns with previous findings that the effects of phytogenic feed additives are often phase-dependent, with more evident responses during later growth stages (Ciftci et al., 2005; Mountzouris et al., 2011). These findings are supported by evidence that phytogenic additives improve digestibility in later growth stages, enhancing gastrointestinal function and nutrient utilization (Hernandez et al., 2004). Since the chicken growth in this study was consistent with that of commercial broilers, as estimated by nonlinear function models (Firas and Al-Samarai, 2015). Moreover, the supplementation trial verified the safety of both fractions in broilers, with no indications of mortality, behavioral disturbances, pathological lesions, and carcass chrematistics. Regarding serum biochemical parameters, no significant effects were detected on serum lipid profile, liver enzymes, or bilirubin levels, indicating that *Z. myriacanthum* byproducts also did not induce metabolic disturbances or hepatotoxicity. Although AD and SR supplementation did not enhance growth performance, the lack of negative effects on overall growth, mortality, and serum biochemical parameters indicates that these fractions are safe for broiler chickens. These results are consistent with previous studies demonstrating non-toxic effects of plant-derived essential oils, particularly those obtained from *Z. bungeanum*, in both laying hens and broilers(Cao et al., 2025; Chen et al., 2024). Although comprehensive toxicological data are available for several Zanthoxylum species, no direct toxicity studies have been reported for *Z. myriacanthum*. Previous investigations on related species (e.g., *Z. chalybeum, Z. zanthoxyloides*, and *Z. heitzii*) indicate that high doses of crude extracts can induce toxicity in rodent models, whereas lower doses and solvent-fractionated extracts generally exhibit reduced or no toxicity (Okagu et al., 2021).

The pH value of meat is an important parameter utilized during the meat processing industries for determining meat quality. Although supplementation with AD and SR significantly influenced pH at 24 h postmortem, the observed values for both breast and thigh meat remained within the normal physiological range reported for broiler meat. Normal broiler meat with red, firm, and nonexudative (RFN) characteristics typically exhibits ultimate pH values of approximately 5.7–6.1, whereas pale, soft, exudative (PSE) meat is associated with values below 5.7 (Barbut et al., 2005; Kovačević et al., 2025). A further variable also influences the physical characteristics of muscle is the meat color, which is known to be altered, either beneficially or adversely, by myoglobin concentration and muscular pH value (Mir et al., 2017). In this study, the increase in breast meat redness were observed with AD and SR supplementation. Additionally, the consumers perceive bright red meat as fresh and of superior quality; nevertheless, pale, discolored, or darkish meat is considered by consumers to be approaching spoilage and of lower quality (Faustman and Cassens, 2007; Mancini and Hunt, 2005). With supplementation of both fractions, thawing loss was reduced, indicating improved water-holding capacity of the poultry meat (Barbut, 2024).These findings are partially consistent with a previous study showing that dietary thyme and rosemary essential oils influence meat color and water-related traits without affecting growth performance (Gumus and Gelen, 2023).

The lower relative liver and intestine weights in the supplemented groups, particularly with AD, despite unchanged performance traits, may indicate metabolic adaptation or improved digestive efficiency. The upregulation of *OCLN* in the AD group suggests reinforcement of tight junction structure and improved epithelial barrier stability, as occludin is a key determinant of tight junction integrity (Suzuki, 2013). This finding aligns with previous reports showing that oregano essential oil supplementation enhances ileal epithelial barrier function in late-phase laying hens (Feng et al., 2021). Whereas the pronounced increase in *GLUT2* expression in the SR group indicates enhanced capacity for transcellular glucose transport, consistent with improved nutrient absorptive function due to increased expression of intestinal sugar transporters (Sadr et al., 2025). Moreover, the increased villus height and VH:CD ratio in the SR group indicate improved intestinal absorptive efficiency, supporting established evidence that such morphological changes are associated with enhanced nutrient utilization in chickens and pigs (Yamauchi, 2007). These findings regarding the effects of SR on nutrient absorption and villus morphology are supported by evidence that appropriate dietary fiber inclusion promotes gastrointestinal development and enhances digestive and absorptive efficiency in poultry (Jha and Mishra, 2021). Furthermore, the correlation patterns suggest a potential mechanistic pathway whereby modulation of intestinal barrier–related gene expression by *Z. myriacanthum* fractions may influence villus morphostructure, which in turn could affect nutrient absorption efficiency and downstream meat quality traits. Collectively, these findings indicate that AD primarily supports barrier function, whereas SR more strongly promotes nutrient transport efficiency in the broiler intestine.

The selective upregulation of *IL-10* in the AD group indicates enhanced anti-inflammatory regulation. Since, IL-10 plays a conserved role in regulating intestinal inflammation in chickens, helping to balance pathogen control with protection of gut tissue integrity (Meunier et al., 2025). This supports mucosal immune homeostasis rather than generalized immunosuppression. In most instances, IL-10 action occurs after the inflammatory response and suppresses further inflammation by regulating the production of proinflammatory cytokines (Arendt et al., 2016). Moreover, the downregulation of the *IL-1β* and *TNF-α* was also observed in the AD and SR supplementation groups. This shift toward reduced expression of pro-inflammatory cytokines suggests attenuation of basal intestinal inflammatory tone, which may help preserve epithelial integrity and reduce energy expenditure associated with immune activation (Al-Sadi et al., 2009). Additionally, previous reports have documented that extracts of *Z. myriacanthum* var. *pubescens* inhibit the production of pro-inflammatory cytokines, including TNF-α and IL-1β, in LPS-activated human monocytic THP-1 cells and significantly suppress nitric oxide production in LPS-stimulated RAW 264.7 macrophages without affecting cell viability (Li et al., 2014; Zhang et al., 2017). Supporting this mechanism, essential oil from *Z. myriacanthum* var. *pubescens* suppresses NF-κB signaling and reduces the expression of pro-inflammatory cytokines (*IL-1β, IL-6*, and *IL-12p35*) from colonic tissue in a dextran sulfate sodium–induced intestinal inflammation mouse model (Ji et al., 2016). Furthermore, a comprehensive review of *Zanthoxylum* species reports that multiple bioactive constituents exhibit anti-inflammatory activity through key signaling pathways, including NF-κB and MAPK, supporting the immunomodulatory potential observed in the present study (Balkrishna et al., 2024).

For microbiome analysis, the shared core microbiota across dietary groups indicates that AD and SR maintained overall microbial stability, while distinct OTUs unique to each group reflect adaptive responses to dietary substrates. The unchanged alpha diversity indicates that AD and SR supplementation did not disrupt overall microbial richness or evenness, suggesting preservation of microbial ecological balance within the cecum. These observations align with ecological theory indicating that microbiome stability arises from balanced microbial interactions that limit positive feedback, allowing community composition to shift while maintaining overall structural stability (Coyte et al., 2015). However, the clustering in the PCoA indicates that different dietary substrates selectively promoted specific microbial communities without changing overall diversity. This pattern suggests that AD and SR supplementation reshaped microbiota composition rather than disrupting community stability. The predominance of *Faecalibacterium, Bacteroides*, and *Alistipes* is consistent with microbiota commonly reported in the ceca of healthy poultry (Aruwa et al., 2021). Moreover, the notable abundance of *Lachnoclostridium* and *Alistipes* has been linked to favorable metabolic profiles and has been reported in Chinese native chickens with higher market weights (Yang et al., 2023). These findings reveal that AD and SR supplementation maintained a microbiota profile typical of healthy chickens. Interestingly, *Bacteroides* was significantly altered in both supplemented groups based on pairwise STAMP comparisons with the control group. Alterations in *Bacteroides* abundance may influence complex carbohydrate fermentation and short-chain fatty acid production in the chicken cecum (Fan et al., 2023). Whereas most differentially abundant genera between the AD and SR groups belonged taxonomically to the phylum *Firmicutes, Dietzia* was classified within *Actinobacteriota.* Notably, Firmicutes constitute a major bacterial phylum involved in butyrate and acetate production (Ali et al., 2022), suggesting potential functional implications for cecal fermentation and energy metabolism. Actinobacteriota are typically present at low abundance in the chicken cecum, comprising only a small proportion of the microbial community compared with the dominant Firmicutes and Bacteroidetes (Rychlik, 2020). Despite their low abundance, they contribute to gut microbiota homeostasis and modulate host immune and metabolic functions (Binda et al., 2018). Collectively, AD and SR selectively modulated microbial composition while maintaining a stable, health-associated cecal ecosystem. Finally, we acknowledge that the small sample size (*n* = 3 per treatment) for the cecal microbiota sequencing is a primary limitation of this study. While this sample size is often utilized in exploratory pilot studies to provide a preliminary overview of microbial shifts, it may lack the statistical power to detect subtle variations or low-abundance taxa due to high individual variability among birds. To minimize this impact, we strictly selected individuals with body weights closest to the group mean for sequencing. However, the current microbiota findings should be interpreted as exploratory. Future research incorporating larger cohorts (*n* = 6–8) and multi-omics approaches is warranted to fully elucidate the complex interactions between *Z. myriacanthum* by-products and the broiler gut ecosystem.

## Conclusions

Dietary supplementation of AD or SR derived from *Z. myriacanthum* at 1,000 mg/kg in broilers was non-toxic and did not negatively affect overall growth performance. Both fractions positively influenced meat quality. In addition, SR improved intestinal villus morphostructure and increased nutrient glucose absorption. Notably, both AD and SR positively modulated broiler pro-, and anti-inflammatory cytokines. The findings suggest that hydrodistillation by-products of *Z. myriacanthum* may serve as attractive and alternative phytogenic feed ingredients for offering practical benefits for meat quality, gut health, and immunomodulation in broiler production.

## Institutional review board statement

The animal study protocol was conducted in strict accordance with the guidelines recommended and ethically approved by the Animal Ethics Committee, Faculty of Agriculture, Chiang Mai University with the protocol number RAGIACUC013/2567.

## Informed consent statement

Written informed consent was obtained for all participants involved in this study.

## Data availability statement

The data presented in this study are available on request from the corresponding author.

## Funding

This work was supported by Chiang Mai University for graduate research under the Graduate Program in Veterinary Science (Grant ID: 651455802).

## CRediT authorship contribution statement

**Yu-Lei Wang:** Writing – review & editing, Writing – original draft, Visualization, Validation, Software, Methodology, Investigation, Formal analysis, Data curation. **Chompunut Lumsangkul:** Supervision, Resources, Methodology, Investigation, Data curation, Conceptualization. **Sarana Rose Sommano:** Supervision, Resources, Methodology, Conceptualization. **Tossapol Moonmanee:** Supervision, Methodology, Conceptualization. **Sureerat Numee:** Methodology, Conceptualization. **Chiao-Hsu Ke:** Validation, Formal analysis, Data curation. **Patipan Hnokaew:** Validation, Data curation. **Kiattisak Huanhong:** Investigation. **Raktham Mektrirat:** Writing – review & editing, Writing – original draft, Visualization, Validation, Supervision, Software, Resources, Project administration, Methodology, Investigation, Funding acquisition, Formal analysis, Data curation, Conceptualization.

## Disclosures

The authors declare that they have no known financial or personal relationships that could have appeared to influence the work reported in this manuscript.
